# Hypercalciuria in Sanjad-Sakati Syndrome: A Retrospective Evaluation of Kidney Involvement Indicators

**DOI:** 10.7759/cureus.102457

**Published:** 2026-01-28

**Authors:** Vina Tresa, Saima Zeb Shaikh, Saima Mehmood, Anwar AL-Omairi, Komal Fatima, Dana Ahmed Al Nabhani, Saif Al Yaarubi

**Affiliations:** 1 Department of Pediatric Nephrology, Sultan Qaboos University Hospital, Muscat, OMN; 2 Department of Pediatric Nephrology, Sindh Institute of Urology and Transplantation, Karachi, PAK; 3 Department of Pediatrics, Tawam Hospital, Al Ain, ARE; 4 Department of Pediatrics and Child Health, The Aga Khan University Hospital, Karachi, PAK; 5 Department of Child Health, Sultan Qaboos University Hospital, Muscat, OMN; 6 Department of Pediatric Endocrinology, Sultan Qaboos University Hospital, Muscat, OMN

**Keywords:** hypercalciuria, hypoparathyroidism, kidney stones, nephrocalcinosis, rare genetic syndrome, sanjad-sakati syndrome

## Abstract

Objective: This study aimed to evaluate the kidney and urinary abnormalities in children diagnosed with Sanjad-Sakati syndrome (SSS).

Methods: This was a retrospective, descriptive hospital-based study performed at the Child Health Department, Sultan Qaboos University Hospital, Oman. The study included all pediatric patients up to the age of 15 years who presented with clinically and/or genetically confirmed SSS from January 2006 to December 2020.

Results: Fifteen patients were enrolled in the study. Hypercalciuria was present in 15 (100%) patients. The majority of children were observed to be less than five years at the time of onset of hypercalciuria (age range from 2 to 14 years). Nephrocalcinosis was observed in 10 (66.7%) patients. Nonobstructive kidney stones were identified in two (13.3%) patients at ages 6 and 11 years. At the last follow-up, nine (60%) had normal kidney function, while four (26.7%) and two (13.3%) patients were observed to have chronic kidney disease stages 1 and 2, respectively. No case of end-stage kidney disease was detected.

Conclusion: These results highlight significant kidney involvement, particularly hypercalciuria and nephrocalcinosis, in patients with SSS, followed by consequent stones and progressive kidney dysfunction, indicating the need for regular kidney surveillance to ensure timely detection and management of evolving kidney disease.

## Introduction

Sanjad-Sakati syndrome (SSS), which is also referred to as hypoparathyroidism-retardation syndrome, was initially described by Sanjad et al. [1]. It is a rare autosomal recessive disorder characterized by hypoparathyroidism, growth failure, distinctive craniofacial dysmorphisms, and intellectual disability. Common manifestations include hypocalcemic seizures during the neonatal period, growth retardation, and developmental delay [2].

SSS is primarily attributed to mutations within the TBCE gene. This gene is located on chromosome 1q42-43 and encodes for the tubulin-specific chaperone E protein [3]. Deficiencies or dysfunctions in the tubulin-specific chaperone E protein have many distinctive features and are usually characterized by developmental delay affecting both physical growth and intellectual milestones, along with distinct facial features such as a small face, deep-set eyes, a beaked nose, and a prominent forehead [4]. These clinical features collectively define the syndrome's recognizable profile.

Hypoparathyroidism is a hallmark feature of the syndrome, often presenting in the neonatal period and persisting into later life, with characteristic clinical features including muscle spasms (tetany) and seizures resulting from decreased calcium levels [5]. The parathyroid hormone (PTH) directly affects bone and kidney function, while its effects on the gastrointestinal tract are indirectly mediated through the modulation of kidney 1,25-dihydroxy vitamin D (1,25(OH)2 D) production [6].

In the kidney, PTH promotes the reabsorption of calcium in the distal portion of the nephron, including the convoluted tubule and the initial segment of the collecting duct. PTH also promotes urinary phosphate excretion in the proximal convoluted tubule by regulating sodium-phosphate cotransporter. This dual action ensures a balanced calcium-phosphate ratio for bone mineralization and metabolic stability. Another important effect of PTH in the kidney is the transcriptional activation of 25-hydroxyvitamin D-1α-hydroxylase, which is required to produce 1,25-dihydroxyvitamin D3 and, thus, indirectly promotes calcium and phosphate reabsorption through the intestine.

In the absence of PTH, as seen in patients with SSS, this finely regulated mechanism is disrupted. Calcium reabsorption in the kidney tubules is impaired, leading to increased urinary calcium excretion (hypercalciuria). Further worsening of this filtered calcium load is contributed to by high-dose calcium and active vitamin D supplementation, which is usually prescribed to these patients to maintain normocalcemia. Hypercalciuria is a well‑recognized risk factor for nephrocalcinosis, nephrolithiasis, and chronic kidney disease (CKD), as documented in multiple cohorts and guidelines addressing chronic hypoparathyroidism [7,8].

While SSS has been extensively studied for its endocrinological and developmental impacts, there is limited research on its kidney manifestations. To our knowledge, this is the first study to focus exclusively on kidney aspects. Although one case report and two previous studies briefly mentioned kidney findings, none have explored them in depth.

This research was planned to study retrospectively the course of kidney involvement in our cohort of SSS who were diagnosed and followed at Sultan Qaboos University Hospital (SQUH). Thus, enhancing awareness of kidney manifestations facilitates early identification and potentially prevents these complications.

## Materials and methods

This was a retrospective, descriptive, hospital-based study carried out at the Child Health Department, SQUH, Oman. Ethical approval for this research was granted by the institutional ethics committee. All pediatric patients with a confirmed diagnosis of SSS between January 2006 and December 2020 were included. Patients with hypoparathyroidism due to causes other than SSS were excluded.

No interventions were applied. The study involved a retrospective review of medical records, laboratory data, and imaging findings. Given the retrospective design, individual informed consent was not applicable. As such, certain methodological details, such as the exact urine collection method, specific imaging criteria for nephrocalcinosis and nephrolithiasis, and the approach used for estimated glomerular filtration rate calculation, were not consistently documented in the medical records. Where possible, we used the available recorded results to define renal and urinary outcomes, and these limitations are acknowledged in the interpretation of the findings.

Patient data were collected through the hospital information system, with all personal identifiers removed to ensure confidentiality. The medical records of all enrolled patients were reviewed from initial presentation to the last follow-up, including details from inpatient and outpatient visits. Information extracted included demographic characteristics, age at diagnosis, clinical outcomes, and detailed kidney findings. Main outcome measures noted were hypercalciuria, nephrocalcinosis, kidney stones, and CKD. Emphasis was placed on age at onset of hypercalciuria, nephrocalcinosis, and kidney stones; kidney ultrasound findings; calcium-to-creatinine ratios; and kidney functions at last follow-up.

Data were compiled in Microsoft Excel (Microsoft Corporation, Redmond, WA) and analyzed using Statistical Package for the Social Sciences (version 23.0; IBM Corp., Armonk, NY). Descriptive statistics were used throughout. Continuous variables such as age and calcium-to-creatinine ratios were presented as means and ranges. Categorical variables, including hypercalciuria, nephrocalcinosis, kidney stones, CKD, gender, and outcome, were reported as frequencies and percentages. No inferential statistical analyses were conducted due to the small sample size and observational nature of the study.

## Results

Table 1 summarizes the demographic and clinical information for the study participants, highlighting their baseline characteristics. A total of 15 patients were enrolled in the study, with ages ranging from 2 to 14 years. The majority of 10 (66.7%) patients were diagnosed with SSS before reaching one year of age. Mortality in our study population was observed to be four (26.7%), and the cause of death was fulminant infections. Three of the patients’ ages at diagnosis were missing in the records.

**Table 1:** Baseline characteristics of patients

Characteristic	Male (n = 6)	Female (n = 9)	Total (n = 15)
Age at enrolment			
<1 year	-	-	-
>1 year	6 (40)	9 (60)	15 (100)
Age at diagnosis			
<1 year	3 (30)	7 (70)	10 (66.7)
>1 year	-	1 (100)	1 (6.7)
Missing data	3 (75)	1 (25)	4 (26.7)
Alive	4 (36.3)	7 (63.6)	11 (73.3)
Dead	2 (50)	2 (50)	4 (26.6)

**Figure 1:**
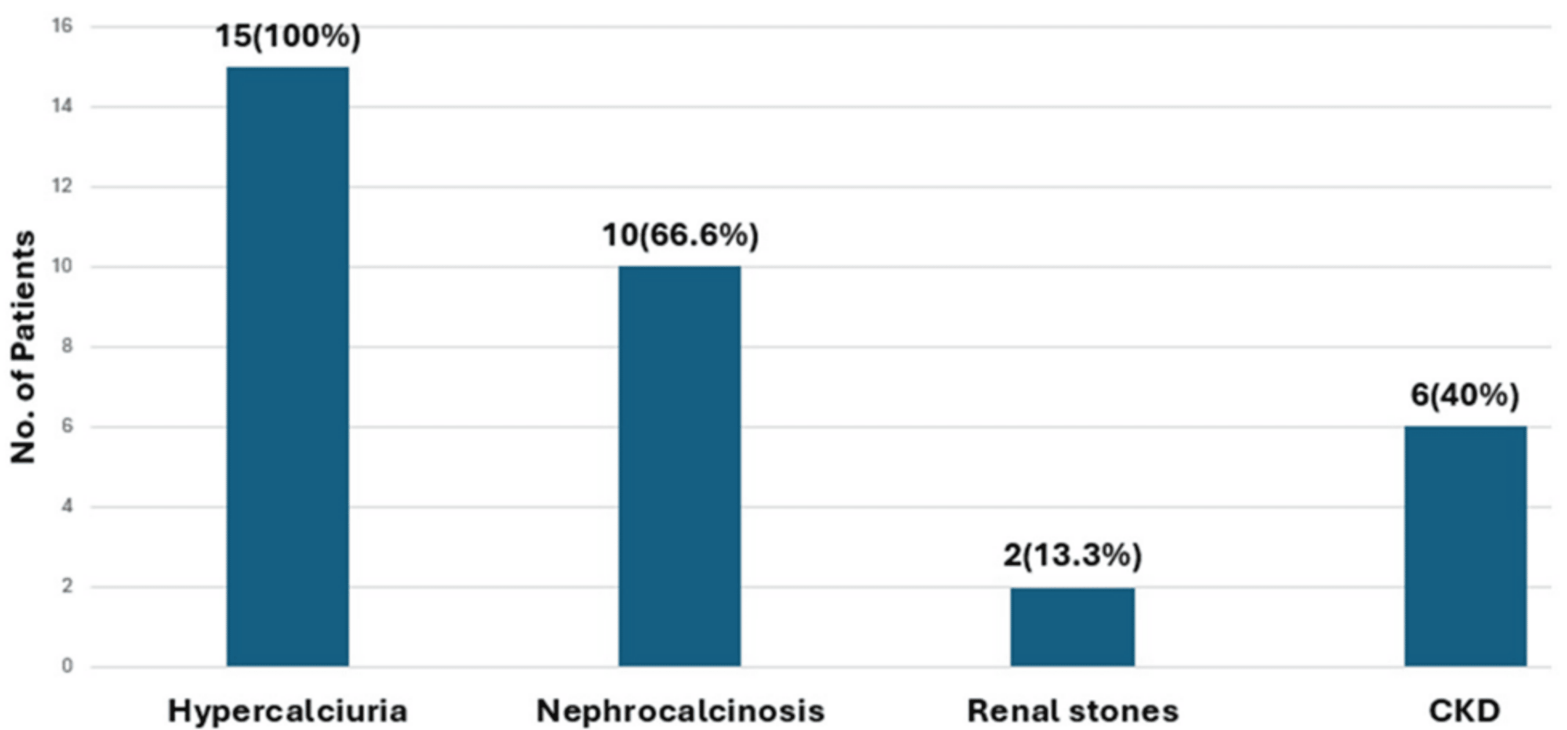
Renal manifestations of SSS CKD: chronic kidney disease; SSS: Sanjad-Sakati syndrome

Figure 1 shows that hypercalciuria emerged as the most prevalent finding, followed by nephrocalcinosis. A smaller proportion of children developed CKD, while nonobstructive kidney stones were the least frequently noted complication.

Table 2 illustrates the timing of kidney manifestations in the cohort. Hypercalciuria and nephrocalcinosis were not detected during infancy and were observed in the majority of patients after the first year of life. Notably, one patient presented with nephrocalcinosis at nine months of age, preceding the onset of hypercalciuria, which occurred at two years of age. The mean calcium-to-creatinine ratio in the cohort was 1.85 (range, 0.18-5.3). Kidney stones were identified in two patients, at ages 6 and 11 years, respectively, and neither of them developed obstructive uropathy.

**Table 2:** Age of onset of different renal manifestations

Age groups	Renal manifestations		
	Hypercalciuria, n (%)	Nephrocalcinosis, n (%)	Renal stones, n (%)
<1 year	-	1 (6.7)	-
>1-5 years	6 (40)	3 (20)	-
>5-10 years	5 (33.3)	3 (20)	1 (50)
>10-15 years	4 (26.7)	3 (20)	1 (50)

Table 3 shows the kidney function status of SSS; nine (60%) patients were noted to have normal kidney function until their last follow-up, and four (26.7%) were observed to have mild kidney dysfunction. The median age at the last follow-up was nine years (range, 4-21 years).

**Table 3:** Renal status on last follow-up

Variables		Gender (n = 15)		Total (n = 15), n (%)
		Male	Female	
Renal functions on last follow-up	Normal	4	5	9 (60)
	CKD-1	2	2	4 (26.7)
	CKD-2	-	2	2 (13.3)

## Discussion

SSS is a hereditary condition that shares features with Kenny-Caffey Syndrome type 1. Both conditions are inherited in an autosomal recessive pattern and are caused by mutations in the tubulin-specific chaperone E gene. Other etiologies of syndromic hypoparathyroidism include several genetic conditions: DiGeorge syndrome, associated with mutations in TBX1 and NEBL; hypoparathyroidism-deafness-renal dysplasia syndrome, linked to mutations in GATA3; X-linked hypoparathyroidism, caused by mutations in SOX3; and Kenny-Caffey syndrome type 2, resulting from mutations in FAM111A [9].

SSS has been most frequently reported from the Arabian Gulf region, particularly Saudi Arabia and Kuwait, where the high prevalence of consanguineous marriages contributes to increased disease occurrence. Although initially thought to be confined to Arab populations, SSS has subsequently been reported in non-Arab populations, including cases from Sudan, India, and Egypt. For example, Arabi et al. described 11 Sudanese children with SSS, and similar reports have emerged from India, Egypt, and Morocco, suggesting that the condition may be more geographically widespread than previously assumed [10-14]. One study estimated the incidence of SSS in Kuwait at 7-18 per 100,000 live births [15].

This study identified kidney abnormalities in patients with SSS, highlighting the consistent presence of hypercalciuria across the cohort. This finding is expected in the context of congenital hypoparathyroidism and the use of high-dose calcium and vitamin D therapy. Hypercalciuria was observed in all patients (100%), with onset after age 1. These findings align with the established pathophysiology of congenital PTH deficiency, where renal calcium loss typically increases in early childhood [16].

Furthermore, chronic hypercalciuria is a significant risk factor for nephrocalcinosis and nephrolithiasis [17]. Thus, hypercalciuria in SSS is not merely a biochemical finding but a consistent, early marker of kidney vulnerability.

Nephrocalcinosis was identified in 10 of 15 patients (66.7%), with a mean age of onset at eight years. Notably, one infant was diagnosed with nephrocalcinosis prior to the onset of measurable hypercalciuria, an observation adding a new perspective as to how kidney involvement may unfold in SSS. This early onset challenges the usual understanding that hypercalciuria comes first and raises the possibility of other alternative pathophysiologic mechanisms. Such observations suggest that children with SSS may have an increased kidney sensitivity to calcium deposition, perhaps due to tubular dysfunction, immature kidney handling of minerals [18], or the effects of high-dose calcium and vitamin D therapy [19]. This emphasizes the need for careful, adjusted therapy guided by close kidney monitoring.

Two patients (13.3%) developed kidney stones at the ages of 6 and 11 years. Notably, no case of obstructive uropathy secondary to nephrolithiasis was identified. Although the incidence of nephrolithiasis appeared to be less frequent in our study population compared to other kidney manifestations, this finding may be attributable to the limited sample size. Furthermore, it emphasizes the importance of ongoing monitoring for kidney complications extending beyond early childhood [20].

By the time of the last follow-up, six of the children had developed mild kidney dysfunction, classified as having CKD stage 1 in four (26.7%) patients and CKD stage 2 in two (13.3%) patients. None of the cases progressed to end-stage kidney disease, which highlights the silent progression of kidney impairment in SSS. In contrast, Kenny-Caffey syndrome, which closely resembles SSS, exhibits CKD primarily due to structural abnormalities, including small kidneys, rather than nephrocalcinosis. These structural defects may lead to reduced concentrating ability and progressive tubulointerstitial damage [21].

This study revealed that early hypercalciuria often precedes nephrocalcinosis, suggesting an opportunity for timely intervention. While conventional treatments focus on managing calcium levels and utilizing active vitamin D analogues, the use of thiazide diuretics in patients with documented hypercalciuria can provide significant improvements, including normalization of serum calcium levels and a reduction in the filtered calcium load [22]. Emerging therapies such as recombinant human PTH may show promise in reducing urinary calcium excretion and preventing kidney complications [19]. However, further research is necessary to fully establish their long-term benefits for kidney preservation.

Limitations

Like many rare disease studies, this one has its limitations. Being retrospective and based on a small, convenience sample, the findings may not fully represent the broader SSS population. The limited number of patients also makes it harder to draw strong conclusions about how kidney issues progress over time. Additionally, potential information bias from incomplete records and variable follow-up assessments may affect the accuracy of the outcomes. Still, these results offer important early insights and future prospects. Further research with a larger sample and extended follow-up is essential to a more comprehensive understanding of kidney involvement in this syndrome.

## Conclusions

We observed hypercalciuria and nephrocalcinosis as an underrecognized yet clinically significant kidney manifestation of SSS progressing to kidney stones and mild kidney dysfunction over time. Greater awareness of renal complications among clinicians caring for these patients is crucial. Regular kidney surveillance is strongly recommended to minimize morbidity in affected children. Further prospective, multicenter studies with higher number of patients are needed to define optimal surveillance strategies and therapeutic approaches aimed at preserving kidney function in SSS.
